# Dynamic Regulation of Extracellular Signal-Regulated Kinase (ERK) by Protein Phosphatase 2A Regulatory Subunit B56γ1 in Nuclei Induces Cell Migration

**DOI:** 10.1371/journal.pone.0063729

**Published:** 2013-05-21

**Authors:** Ei Kawahara, Shiori Maenaka, Eri Shimada, Yoshihiro Nishimura, Hiroshi Sakurai

**Affiliations:** Department of Clinical Laboratory Science, Kanazawa University, Kanazawa, Japan; University of Leuven (KU Leuven), Faculty of Medicine, Belgium

## Abstract

Extracellular signal-regulated kinase (ERK) signalling plays a central role in various biological processes, including cell migration, but it remains unknown what factors directly regulate the strength and duration of ERK activation. We found that, among the B56 family of protein phosphatase 2A (PP2A) regulatory subunits, B56γ1 suppressed EGF-induced cell migration on collagen, bound to phosphorylated-ERK, and dephosphorylated ERK, whereas B56α1 and B56β1 did not. B56γ1 was immunolocalized in nuclei. The IER3 protein was immediately highly expressed in response to costimulation of cells with EGF and collagen. Knockdown of IER3 inhibited cell migration and enhanced dephosphorylation of ERK. Analysis of the time course of PP2A-B56γ1 activity following the costimulation showed an immediate loss of phosphatase activity, followed by a rapid increase in activity, and this activity then remained at a stable level that was lower than the original level. Our results indicate that the strength and duration of the nuclear ERK activation signal that is initially induced by ERK kinase (MEK) are determined at least in part by modulation of the phosphatase activity of PP2A-B56γ1 through two independent pathways.

## Introduction

Extracellular signal-regulated kinase (ERK) signalling plays a central role in basic biological processes of cells. A growth factor initiates a cell signal by binding to its cell surface receptor, and this is followed by activation of ERK via phosphorylation by ERK kinase (MEK) at threonine and tyrosine residues in the TEY motif (pTpYERK) [1]–[3]. Subsequently, ERK phosphorylates about 200 distinct substrates, ranging from transcription factors to cytoskeletal proteins, and from protein kinases to phosphatases [4]. The great diversity of ERK substrates is consistent with the diverse effects of ERK on cellular functions [5], [6]. The question of how a single event is chosen among the variety of ERK-induced functions has been extensively studied, and it appears that particular cellular responses require a specific strength and duration of ERK activation [7], [8]. Initially, it was thought that sustained ERK activation caused cell differentiation, while transient ERK activation led not to proliferation, but to migration [9], [10]. However, cell proliferation also requires sustained ERK activation associated with integrin-mediated anchorage [11], which induces a gradual increase in ERK activation that is subsequently sustained [12]. A growth factor initially activates ERK rapidly and transiently [13], and then synergistic signalling from the anchorage and the growth factor induces robust and sustained ERK activation [11], [14]. Thus, all anchorage-dependent cellular events, including migration [15]–[18], proliferation [11], [19], differentiation [9] and survival [20], induced by a growth factor require sustained activation of ERK [21].

The choice of cellular outcome is presumably influenced by differences of stimulus intensity and duration or growth factor concentration. It has been shown that low concentrations of a growth factor induce cell proliferation but not cell migration, whereas higher concentrations induce cell migration on a matrix and inhibit cell proliferation [16], [22]. Thus, subtle differences of growth factor stimulation produce differences in the strength and duration of ERK signalling, leading to specific biological outcomes through distinct mechanisms involving efficient ERK sensors, including phosphorylation of c-FOS protein [23]. The strength of ERK activation is mainly determined by the balance of activities of MEK and constitutively active phosphatases. It is also modulated by inactivating signals, which could originate from cross-talk with a parallel signal, a negative feedback signal or a positive feedback signal. Thus, the strength and duration of ERK activation appear to be precisely regulated by a complex network of factors, many of which remain to be fully defined.

It seems likely that phosphatases are important modulators of ERK activation. One or two phosphorylated sites in dual-phosphorylated ERK could be dephosphorylated and inactivated by dual-specific mitogen-activated protein kinase (MAPK) phosphatases (MKPs) [24], tyrosine phosphatases in specialized cells [1], [2], and Ser/Thr protein phosphatases [1], [25], [26]. MKPs are well-known phosphatases that specifically dephosphorylate pTpYERK through a posttranscriptional negative feedback mechanism [24], [27], which would be involved in halting ERK-induced cellular events. A protein-tyrosine phosphatase (PTP) acts as an important regulator of ERK in hematopoietic cells [28], although PTPs are dispensable for ERK regulation in other cells [29]. On the other hand, accumulating evidence indicates that PP2A has complex inhibitory and stimulatory effects on growth factor- and/or adhesion-induced signalling, in particular the ERK cascade. PP2A substrates include receptor tyrosine kinases [30], [31], receptor-associated adaptor proteins [32], and all three kinases of the ERK cascade, Raf1 [33]–[35], MEK [36], [37], and ERK [25], [38], [39]. PP2A influences tumor progression [40]–[42]. It is also well-known to be involved in one of the most important anchorage-dependent cellular events, i.e., cell motility [43], [44], which is in turn involved in cancer invasion and metastasis.

The great diversity of PP2A functions has been ascribed to the diversity of its B regulatory subunits [42]. The B regulatory subunits of PP2A heterotrimer are divided to B, B56 (B′) and B′′ families. Each family consists of several gene products and splicing variants, and their individual functions and substrate specificities have been partly identified. Bα- and Bδ-containing PP2A promote activation of ERK by dephosphorylating the Raf1 inhibitory phosphorylation site [35]. It has also been found that PP2A with members of the B56 family can dephosphorylate ERK directly, and that this action is related to cell migration. Letourneux et al. [39] and Rocher et al. [45] reported that PP2A-B56β1, B56β2, B56γ1 and B56γ2 dephosphorylate ERK directly, but they did not identify a corresponding cellular event. However, it was also reported that B56γ1 inhibited locomotion through dephosphorylation of paxillin or mdm2 [46], [47]. p53 protein was also reported to be a substrate of PP2A-B56γ1 and -B56γ3 [48], [49]. PP2A-B56β1 dephosphorylates Pim-1 [50], TrkA [31], tyrosine hydroxylase [51], Akt1 [52], [53] and Emi2 [54]. In addition, B56α targets cMyc, APC, ankyrin B [55] and SK1 [56], and PP2A-B56γ3 dephosphorylates ChK2 [57]. Thus, the functions of the B56 family members appear to be quite redundant, and it seems likely that the central regulatory mechanism of ERK activation remains to be fully defined.

In this work, we aimed to investigate which subunits of the B56 family are directly involved in ERK activation leading to cell migration. To address this question, we carried out costimulation experiments with EGF and collagen, using Calu1 lung carcinoma cells and mouse Swiss 3T3 fibroblasts. We found that ERK in nuclei is dephosphorylated only by PP2A-B56γ1. Furthermore, we found two temporally distinct kinetic curves involving PP2A-B56γ1; initial loss of activity following stimulation, then sustained low-level activation caused by prompt synthesis of IER3. Our results indicate that the fidelity of the strength and duration of the nuclear ERK activation associated with cell migration is maintained by dynamic regulation mediated by PP2A-B56γ1.

## Materials and Methods

### Cells

Calu1 lung squamous cell carcinoma line [58] was kindly provided by Dr Takashi Takahashi (Aichi Cancer Institute, Japan), but is also available through American Type Culture Collection. OSC19 oral squamous cell carcinoma line [59] was kindly provided by Dr Yokoi (Sapporo Medical University, Japan). Mouse Swiss 3T3 fibroblasts [59] and a variety of human squamous cell carcinoma lines including HSC3 oral cell carcinoma [60], EBC1 lung carcinoma, LK2 lung carcinoma and A431 esophageal carcinoma [16] were obtained from Japanese Collection of Research Bioresources (Osaka, Japan). The cells of subconfluent cultures, maintained in MEM containing 10% fetal bovine serum, were harvested with trypsin, and then the trypsin was inactivated with soybean trypsin inhibitor (Sigma, St Louis, MO, USA). The cells were washed with serum-free MEM containing 0.5% heat-denatured bovine serum albumin (Sigma) and were used for experiments.

### Migration Assay

Cell migration assay was performed in 24-well Transwell chambers containing a polycarbonate membrane with a pore size of 8 µm (Corning, Acton, MA, USA) [61]. The undersurface of the membrane was coated with 10 µg/ml type I collagen solution in PBS. Aliquots of 50,000 cells were treated with or without EGF. Cells were treated with PD98059 or cycloheximide for 30 min prior to EGF stimulation, when necessary. The cells were loaded onto membranes in quadruplicate, and incubated in a CO_2_ incubator at 37°C. The cells that migrated on the collagen-coated surface were stained with 2% crystal violet. The membranes were washed with water, the dye was eluted with 10% acetic acid, and the optical density of the eluate at 620 nm was measured. When the number of migrated cells was too small for colorimetric assay, the cells were counted directly under a microscope.

### Plasmids

pCEP plasmids containing B56α1, B56β1 or B56γ1 cDNA with a 4HA tag (pCEP4HAB56α1, pCEP4HAB56β1, pCEP4HAB56γ1) were provided by Dr David Virshup [62]. Mouse B56γ1 containing vector (pCX4bsr-B56γ1) was provided by Dr Ito [63].

### DNA Transfection

The vectors containing B56 were transfected into cells using Lipofectamine 2000 (Invitrogen). pCEP4HAB56α1, pCEP4HAB56β1 and pCEP4HAB56γ1 plasmids were used to transfect Calu1 cells and pCX4bsr-B56γ1 to transfect mouse Swiss 3T3 cells. The transfected cells were selected with hygromycin or blasticidin for 1 month and stable cell lines were established. The pCEP4HAB56β1 and pCEP4HAB56γ1 plasmids were also used to transiently transfect Calu1 cells in conjunction with Lipofectamine 2000, and the transfected cells were used for experiments 24 hour later.

### RNAi

To knock down a gene, double-stranded short interference RNA (d-siRNA) was generated using a Block-iT RNAi TOPO Transcription Kit and a Block-iT Dicer RNAi Kit (Invitrogen). A sequence covering IER3 cDNA (470 bp) was amplified by RT-PCR using a set of primers. The resultant PCR product was joined to a linker containing a T7 RNA polymerase-recognizing sequence. From the linked sequence a sense template and an antisense template were amplified with the same set of primers and T7 polymerase specific primer. Each DNA template was converted to single-stranded RNA using T7 RNA polymerase. Sense RNA and antisense RNA were annealed to generate double-stranded RNA. The double-stranded RNA was treated with dicer. The resultant 20–23 bp d-siRNA was transfected into Calu1 cells with Lipofectamine 2000. Gene-specific mRNA expression was measured by a real-time PCR system (ABI PRISM 7000 or Viia7, Applied Biosystems, Foster City, CA, USA), and the specificity and efficiency of knockdown were calculated. For mock RNAi an intron sequence of β5 integrin gene [64], which is unrelated to the genes examined in the present study, was used.

### Western Blotting

Cells were lysed with SDS sample buffer (625 mM Tris-HCl buffer, pH 6.8, 2% SDS, 10% glycerol, 01% bromophenol blue) with or without 6% mercaptoethanol. Samples were subjected to 10–13% SDS-PAGE. The samples run in the gel were transferred onto polyvinylidene fluoride membranes (Amersham Hybond-P, GE Healthcare, Buckinghamshire, UK), and reacted with anti-phospho-ERK1/2 (Thr^202^/Tyr^204^) antibodies (Cell Signaling Technology, Danvers, MA), anti-phospho-ERK1/2 (Tyr^204^) antibodies (Santa Cruz Biotechnology, Santa Cruz, CA, USA), anti-PP2A-Cα/βantibodies (Santa Cruz Biotechnology), anti-actin antibodies (Sigma), anti-phospho-MEK (Ser^218^/Se^r222^), anti-IER3 antibodies (Abnova, Walnut, CA, USA), or anti-HA antibodies (Roche, Manheim, Germany). Then, peroxidase-labeled anti-rabbit IgG antibodies (Cell Signaling Technology), peroxidase-labeled anti-rat antibodies (Cell Signaling Technology) or peroxidase-labeled anti-goat IgG antibodies (Santa Cruz Biotechnology) were applied. Visualisation was performed using a chemiluminescence system (ECL Western Blotting Detection Reagents, Amersham Biosciences, Buckinghamshire, UK). To compare protein levels, the lysates were run in a single polyacrylamide gel and band densities were determined by densitometric analysis.

### Dot Blotting

Quantification of IER3 protein was examined by dot blot analysis. Cells were lysed with SDS sample buffer or lysis buffer. Samples of equal volume were applied in triplicate, dried and crosslinked to PVDF membrane by an ultraviolet crosslinker (UV stratalinker 1800, Stratagene, La Jolla, CA, USA). The membrane was reacted with anti-IER3 antibodies and peroxidase-labelled anti-rabbit IgG antibodies. Chemiluminescence reaction was performed using ECL Western Blotting Detection Reagent and evaluated with an image analyzer (Lumi-Imager F1, Roche Diagnostics). Protein concentrations in the samples were measured using RC-DC Protein Assay. A sample with maximum protein concentration was serially diluted to prepare a standard curve so that relative amounts could be obtained. The amounts of IER3 determined from the standard curve were calibrated with the protein concentrations of the samples.

### Pulldown Assay

HA-B56 transfected Calu1 cells were dissolved in lysis buffer (50 mM Tris-HCl buffer, pH 7.4/150 mM NaCl/1% NP-40/0.25% sodium deoxycholate/1 mM phenylmethylsulfonyl fluoride/1 µg/ml leupeptin/2 µg/ml aprotinin/10 mM mercaptoethanol). Proteins in the cell lysates were immunoprecipitated with protein G Sepharose (Amersham Biosciences) and specific antibodies. The precipitate was washed and SDS-sample buffer with or without mercaptoethanol was added. Protein of interest in the immune complex was detected by western blotting. As a negative control, Protein G Sepharose was suspended in cell lysate without the specific antibodies.

### In vitro Immune Complex Phosphatase Assay

To quantify PP2A-B56γ1 phosphatase activity, a fluorometric phosphatase assay [65] was used. Calu1-B56γ1 cells, which were stimulated with EGF and loaded onto a collagen-coated plate for a suitable period, were lysed with lysis buffer. The protein concentration in the supernatant was measured, and samples containing equal amounts of protein were used. B56γ1 was immunoprecipitated from the lysate with anti-HA antibodies and protein G Sepharose. The immobilized B56γ1 complex was reacted with 4-methylumbelliferyl phosphate (Wako Pure Chemical Industries, Osaka, Japan) in phosphatase buffer (50 mM Tris-HCl, pH 7.5/1 mM MgCl_2_/1 mM EGTA), and the fluorescence intensity of the released 4-methylumbelliferone was measured with an emission wavelength of 450 nm and excitation wavelength of 360 nm using a microplate fluorometer (FluoDia T70, Photon Technology International, Birmingham, NJ, USA).

To qualify phosphatase activity of PP2A-B56 on pTpYERK, B56α1, B56β1 and B56γ1 were immunoprecipitated with anti-HA antibodies, and then immobilized on Protein G Sepharose. Crude cell lysate containing pTpYERK after stimulation with EGF was used as a substrate. Immobilized B56 immune complexes were suspended in the crude cell lysate. As a negative control, Protein G Sepharose without immune complex was reacted with the crude cell lysate.

### Protein Assay

Since cells placed on a collagen-coated plate for less than 5 min were hard to fully collect, protein concentrations were measured using a protein assay kit (RC-DC Protein Assay, Bio-Rad, Hercules, CA, USA) and samples with equal protein amounts were used for western blotting, dot blotting and phosphatase assay.

### Extraction of Nuclear Protein

Cytoplasmic extraction buffer (120 mM KCl/20 mM Tris-HCl buffer, pH 7.9/2 mM EDTA/2 mM dithiothreitol/1 mM phenylmethylsulfonyl fluoride/1 µg/ml leupeptin/2 µg/ml aprotinin) with NP-40 was added to cells. Preipitated nuclei after centrifugation were washed with cytoplasmic extraction buffer. Then, nuclear extraction buffer (40 mM Tris-HCl buffer, pH 7.9/800 mM NaCl, 30 mM EDTA/50% glycerol) was added, vortexed and centrifuged. The resultant supernatant was used for analysis of nuclear protein.

### Immunofluorescence

Glass slides with 8 plastic chambers (Lab-Tek II Chamber Slides, Nunc, Rochester, NY) were coated with 10 µg/ml type I collagen solution. Aliquots of 5,000 cells were loaded into each well and the slide was placed in a CO_2_ incubator for 30 min, and then the cells were stimulated with EGF or not stimulated. The cells were fixed with 3% paraformaldehyde, permeabilized with 0.01% Triton X-100, blocked with 2% BSA, and then incubated with anti-HA antibodies, anti-phospho-ERK1/2 (Thr^202^Tyr^204^) antibodies, anti-mouse B56ΖΖΖ1 antibodies (provided by Dr Nojima), or anti-actin antibodies. As secondary antibodies, Alexa Fluor 488-labeled anti-rabbit antibodies, Alexa Fluor 488-labeled anti-rat IgG antibodies, or Alexa Fluor 594-labeled anti-rabbit antibodies (Molecular Probes, Eugene, OR) were used. Immunofluorescence was observed under a conventional fluorescence microscope (BX50, Olympus, Tokyo, Japan) or a confocal laser scanning microscope (LSM510, Carl Zeiss, Munich, Germany). Nucei were counterstained with Hoechst33258 (Dojindo, Kumamoto, Japan).

### Statistics

Student’s t-test was used for comparison of the averages between two groups. For multiple comparisons, one- or two-way analysis of variance was used and for the subsequent comparison between two groups among them, Scheffe’s multiple comparison was used.

## Results

### Cell Migration Requires Intense and Sustained Activation of ERK

**Figure 1:**
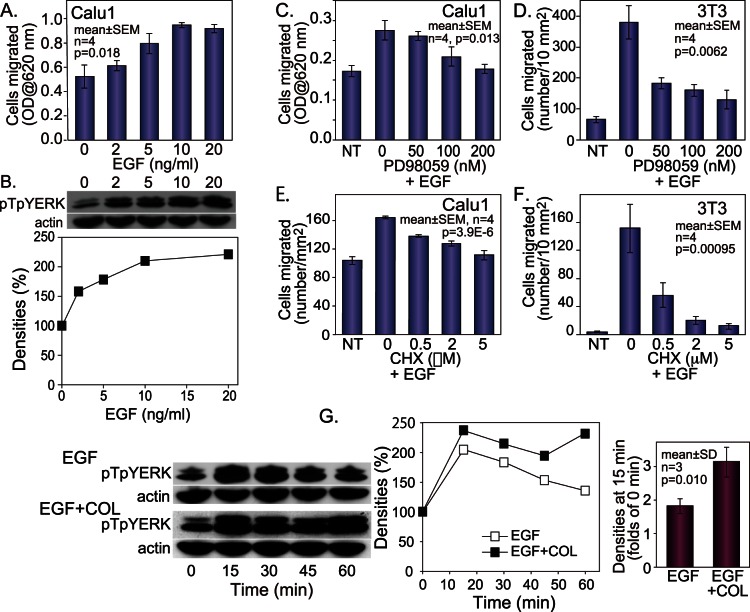
EGF- and collagen-stimulated cell migration and ERK phosphorylation. (A, C–F) Migration assay on collagen using Calu1 carcinoma cells (A, C, E) or Swiss 3T3 fibroblasts (D, F). The cells were allowed to migrate for 5 hours (C, E) or overnight (A, D, F). (A) The dose-dependent effect of EGF on Calu1 cell migration. (B) The dose-dependent effect of EGF on threonine- and tyrosine-phosphorylation of ERK1/2 (pTpYERK) in Calu1 cells. The densities of the bands were measured and plotted on the line chart. (C, D) The effect of PD98059 on EGF-induced cell migration. (E, F) The effect of cycloheximide (CHX) on EGF-induced migration. (G) Kinetics of pTpYERK. Calu1 cells were treated with 10 ng/ml EGF, and the cells were kept in suspension (EGF) or loaded on collagen-coated plates (EGF+COL). The densities of the bands were measured and plotted on the line charts. The levels at 0 min and 15 min were determined by densitometric analysis of samples run in the same gel. One way analysis of variance (A, C, D, E, F) or Student’s t-test (G) was used for statistical analysis.

Migratory activities of various cultured cell lines on collagen were examined using a 5-hour or overnight migration assay. Both Calu1 cells of human lung carcinoma origin and Swiss 3T3 cells of mouse fetal fibroblast origin migrated in response to EGF in this assay system (Figure 1A, F). We further examined a variety of squamous carcinoma cells, including A431 esophageal carcinoma, EBC1 lung carcinoma, LK2 lung carcinoma, OSC19 oral carcinoma and HSC3 oral carcinoma cells, and found that all of them migrated similarly in response to EGF in this assay. The level of pTpYERK in a suspension of Calu1 cells, as estimated by western blotting, was also increased dose-dependently by EGF (Figure 1B). The minimal concentration of EGF that induced both the maximum cell migration and the highest phosphorylation of ERK was 10 ng/ml (Figure 1A, B). Therefore, 10 ng/ml EGF was used for all subsequent experiments in the present study.

The EGF-induced migration of Calu1 cells and 3T3 cells was inhibited by the MEK inhibitor PD98059 (Figure 1C, D), indicating that the EGF-induced migration on collagen is mediated by ERK. The protein synthesis inhibitor cycloheximide also inhibited the EGF-induced migration of Calu1 cells (Figure 1E) and 3T3 cells (Figure 1F) in a dose-dependent manner, indicating that this migration requires de novo protein synthesis.

When EGF was added to a suspension of Calu1 cells (EGF-only group), the level of pTpYERK was increased, reaching the maximum 15 min later, and then the ERK phosphorylation level continuously decreased until 60 min (Figure 1G). When the cells were stimulated with EGF and then immediately loaded onto collagen-coated dishes (EGF+COL), the level of pTpYERK was also increased at 15 min, but this level of phosphorylation then persisted until 60 minutes (Figure 1G). The levels of pTpYERK phosphorylation at 0 min and 15 min under these two different conditions were determined by densitometric analysis of samples run in the same gel. This analysis showed that the level of pTpYERK at 15 min under the EGF+COL condition was significantly higher than that under the EGF-only condition. Thus, both intense and sustained activation of ERK induced by EGF+COL stimulation are required for cell migration.

### Overexpression of B56γ1 Reduces Both EGF-induced Cell Migration and Phosphorylation of ERK

**Figure 2:**
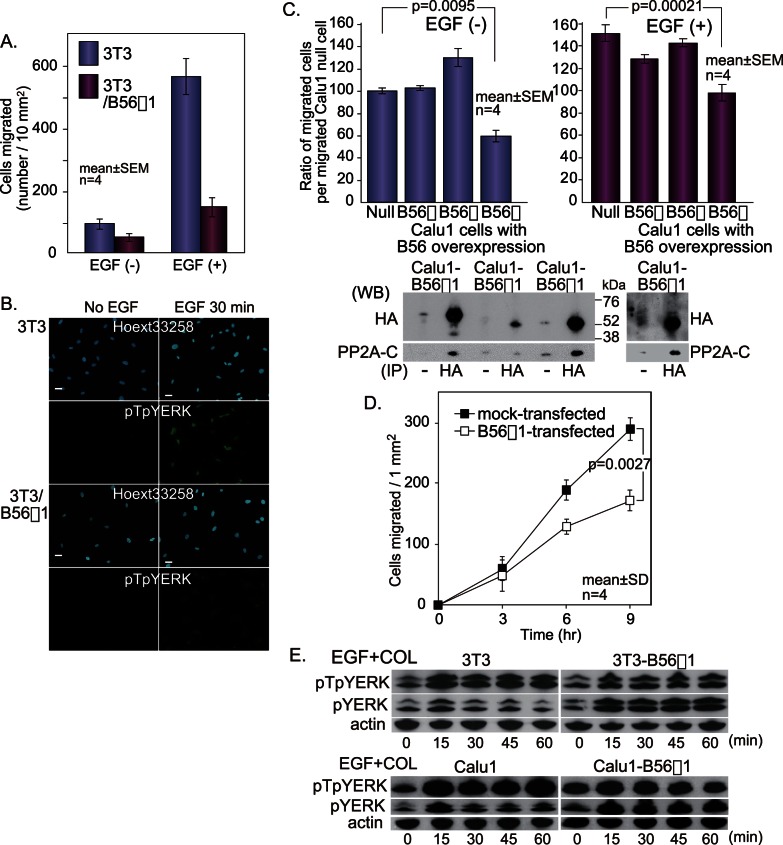
B56γ1 overexpression inhibits both cell migration and ERK phosphorylation. (A) The effect of B56γ1 overexpression on migration of 3T3 cells. (B) The effect of B56γ1 overexpression on ERK phosphorylation in 3T3 cells. Bars, 10 µm. (C) The effects of B56 overexpression on migration using permanently transfected Calu1 cells. Migration of Calu1 cells, which were permanently transfected with pCEPB56α1, pCEPB56β1 or pCEPB56γ1, was compared with that of untransfected Calu1 cells (null). B56 protein in each cell line (2×10^6^ cells) is shown by western blotting with anti-HA antibodies following to immunoprecipitation with anti-HA antibodies, and coprecipitated C subunit is shown by western blotting with anti-PP2A-C subunit antibodies. To detect binding of C subunit to B56β1 protein in B56β1-transfected Calu1 cells, 6×10^6^ cells were used and the blot is shown in the right. (D) The effects of B56 overexpression on migration using transiently transfected Calu1 cells. Migration of Calu1 cells, which were transiently transfected with pCEPB56γ1, was compared with that of Calu1 cells, which were transiently transfected with empty plasmids. (E) The effect of B56γ1 overexpression on kinetics of pTpYERK and pYERK after costimulation in Calu1 cells. Scheffe’s multiple comparison after one way analysis of variance (C) or two way analysis of variance (D) was used for statistical analysis.

To determine what factors, other than MEK, might induce or maintain the intense and sustained activation of ERK following EGF treatment, we analysed the potential involvement of the B56 family of PP2A heterotrimers. The mouse B56γ1 cDNA-containing plasmid pCX4bsr was transfected into 3T3 cells (3T3-B56γ1). The resulting overexpression of B56γ1 led to inhibition of EGF-induced migration of the cells (Figure 2A). The effect of B56γ1 overexpression on ERK phosphorylation was also examined using immunofluorescence assay. 3T3-B56γ1 cells and 3T3 cells were stimulated with EGF and immediately loaded onto collagen-coated glass slides, and then ERK phosphorylation was analysed. The immunofluorescence signal of pTpYERK in 3T3-B56γ1 cells after EGF stimulation was weaker than that in 3T3 cells (Figure 2B).

pCEP plasmids containing B56α1, B56β1 or B56γ1 were permanently transfected into Calu1 cells (pCalu1-B56α1, pCalu1-B56β1, pCalu1-B56γ1). Since all carcinoma cell lines we examined migrated well even when EGF was not added, we examined the effect of B56γ1 overexpression on migration of Calu1 cells in the presence and absence of EGF. pCalu1-B56γ1 cells showed significantly less migration on collagen than did Calu1 cells, both when the cells were stimulated with EGF and when they were not EGF-stimulated (Figure 2C). The pCalu1-B56α1 cells and pCalu1-B56β1 cells migrated similarly to Calu1 cells with or without EGF (Figure 2C). To know if the overexpressed B56 protein forms a complex with C catalytic subunit, pulldown assay was performed. Coprecipitation of C subunit with B56α1 and B56γ1 was detected, and that with B56β1 was not detected when cells with the same count were used (Figure 2C). Coprecipitation of C subunit with B56β1 was detected after the similar protein level of B56β1 to those of B56α1 and B56γ1 was used (Figure 2C).

We also examined the migratory activities of Calu1 cells transiently transfected with pCEP plasmids containing B56γ1 (tCalu-mock). The migratory activity of the transfected cells was compared to that of Calu1 cells that had been transiently transfected with empty pCEP plasmids (tCalu1-mock). The assay showed that the migration of tCalu1-B56γ1 cells was significantly lower than that of tCalu1-mock cells (Figure 2D).

Western blotting indicated that the level of pTpYERK in 3T3-B56γ1 cells after EGF+COL stimulation was slightly lower than that in 3T3 cells (Figure 2E). Analysis of the level of ERK that was phosphorylated only at the tyrosine residue and not at the threonine residue (pYERK), which was assessed using a specific antibody, yielded interesting results. The intensity of the pYERK band increased at 15 min after costimulation with EGF+COL, but then decreased to the original level within 30 min in 3T3 cells. The change was more marked after costimulation of 3T3-B56γ1 cells than after co-stimulation of 3T3 cells (Figure 2E). Similar results were obtained when the same experiment was performed using Calu1 cells (Figure 2E). All of these results suggested that pTpYERK is dephosphorylated (inactivated/inhibited) by PP2A-B56γ1, a serine/threonine phosphatase that dephosphorylates phospho-threonine in the TEY motif.

### PP2A-B56γ1 Dephosphorylates ERK in Nuclei

**Figure 3:**
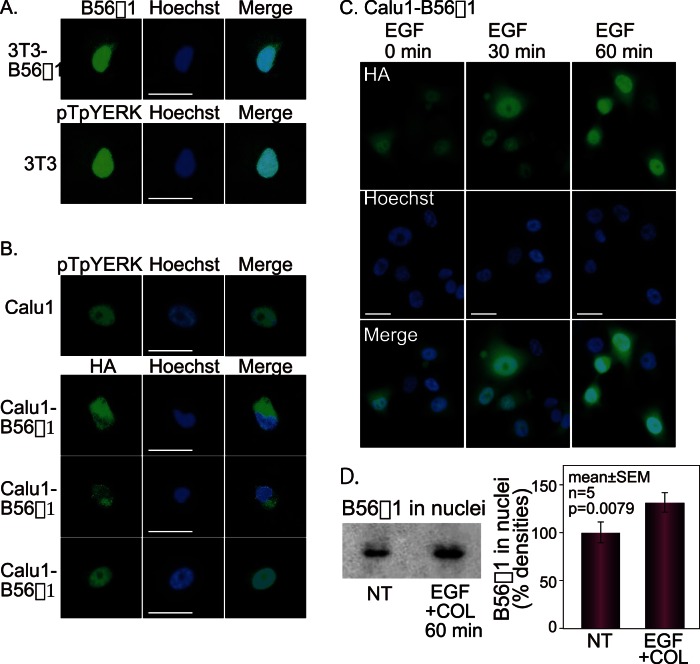
Nuclear localization of pTpYERK and B56γ1. (A) Immunolocalization of B56γ1 in 3T3-B56γ1 cells and pTpYERK in 3T3 cells stimulated with EGF on collagen was analyzed using a confocal laser scanning microscope. To detect mouse B56γ1 protein anti-mouse B56γ1 antibodies were used. (B) Immunolocalization of pTpYERK, B56α1, B56β1 and B56γ1 in Calu1 or B56-transfected Calu1 cells stimulated with EGF on collagen. HA-tagged B56 proteins were detected using anti-HA antibodies. Nuclear or perinuclear immunolocalization was detected by a confocal laser scanning microscope. (C) The time course of B56γ1 in nuclei of B56γ1-transfected Calu1 cells on collagen after stimulation with EGF using a conventional fluorescence photomicroscope. Nuclei were stained with Hoechst 33258. Bars, 10 µm. (D) B56γ1 protein in nuclei. EGF was added to suspension of B56γ1-transfected Calu1 cells, and the cells were immediately loaded on a collagen-coated plastic plate. Extracted nuclear protein from the cells with no treatment (NT) and both with EGF and collagen (EGF+COL) was used for western blot analysis using anti-HA antibodies. Paired t-test was used for statistical analysis.

Possible colocalization of B56 proteins and pTpYERK within cells was examined by immunofluorescence. All of the cell lines analyzed were treated with EGF and placed on collagen for 30 min, followed by immunofluorescence analysis of protein localization. Using a conventional immunofluorescence microscope, B56γ1 in 3T3-B56γ1 cells, detected with anti-mouse B56γ1 antibodies and fluorescence-labelled secondary antibodies, appeared to be localized in both the nuclei and the perinuclear area. Since non-specific fluorescence in the perinuclear area can be an issue in conventional fluorescence microscopy, a confocal laser scanning microscope was next used to examine fluorescence in thin tissue slices. In serial 1-µm-thick sections, B56γ1 in 3T3-B56γ1 cells and pTpYERK in 3T3 cells were clearly localized in nuclei, and no fluorescence signal was observed in the perinuclear region (Figure 3A). pTpYERK immunofluorescence was also detected in the nuclei of Calu1 cells using a confocal laser scanning microscope (Figure 3A). The subcellular localization of B56α1, B56β1, and B56γ1 was further examined by immunofluorescence analysis of permanently B56-transfected Calu1 cells, using anti-HA antibodies. Both B56α1 and B56β1 were localized in the perinuclear area (Figure 3B). B56γ1 was localized only in nuclei and was not seen in any other subcellular localization (Figure 3B).

Immunolocalization of B56γ1 was also examined in the presence and the absence of EGF in pCalu1-B56γ1 cells on collagen. Immunofluorescence of B56γ1 was not clearly observed when the cells were not stimulated with EGF, but was detected in the nuclei at 30 to 60 min after EGF stimulation (Figure 3C). Quantitatively, western blot analysis in nuclear protein extracted from Calu1-B56γ1 cells using anti-HA antibodies showed increase in B56γ1 protein in nuclei after cells were treated with EGF and loaded on collagen (Fig. 3D).

**Figure 4:**
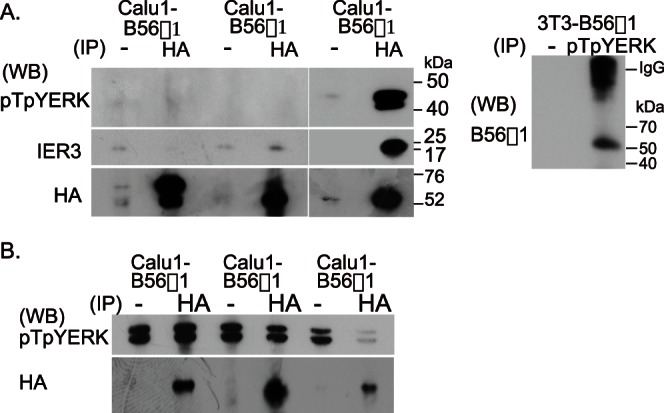
PP2A-B56γ1 binds to and dephosphorylates pTpYERK. (A) Pull down assay. HA-tagged B56 protein in B56-transfected Calu1 cells was immunoprecipitated with anti-HA antibodies. pTpYERK, IER3 and B56s in the immune complex were detected by western blotting. Similarly, the complex immunoprecipitated from 3T3-B56γ1 cells with rabbit anti-pTpYERK antibodies was run in polyacrylamide gel under non-reducing conditions and B56γ1 was detected by western blotting using rabbit anti-mouse B56γ1 antibodies and HRP-labeled anti-rabbit IgG antibodies. (B) Immune complex phosphatase assay. Immobilized PP2A trimer co-immunoprecipitated with anti-HA antibodies on protein G-Sepharose beads was used as an enzyme and pTpYERK-rich cell lysate of Calu1 cells stimulated with EGF was used as a substrate. Beads suspensions were incubated for 30 min in the crude substrate solution. pTpYERK in the reacted lysate was detected by western blotting. Immunoprecipitated enzyme was quantitated by western blotting using rat anti-HA antibodies.

HA-B56 proteins were each immunoprecipitated with anti-HA antibodies from lysates of permanently B56-transfected Call1 cells, and co-precipitating endogenous pTpYERK was analysed by western blotting using anti-pTpYERK antibodies. This experiment showed that B56α1 and B56β1 did not bind to pTpYERK, but that B56γ1 did bind to pTpYERK (Figure 4A). B56γ1 protein was found to co-precipitate with pTpYERK (Figure 4A). To determine the ability of PP2A associated with different B56 subunits to dephosphorylate pTpYERK, in vitro immune complex phosphatase assays were performed. PP2A that co-precipitated with HA-tagged B56 proteins from each pCalu1-B56 cell line was incubated with pTpYERK-rich cell lysates, i.e., lysates of Calu1 cells that had been stimulated with EGF for 30 min. The effect of this incubation on pTpYERK phosphorylation was analysed by western blotting. This experiment showed that PP2A-B56γ1 dephosphorylated pTpYERK, but that PP2A-B56α1 and PP2A-B56β1 did not (Figure 4B), although the amounts of the immunoprecipitated B56s varied somewhat (Figure 4B).

### IER3 Induces Cell Migration via Sustained Activation of ERK

**Figure 5:**
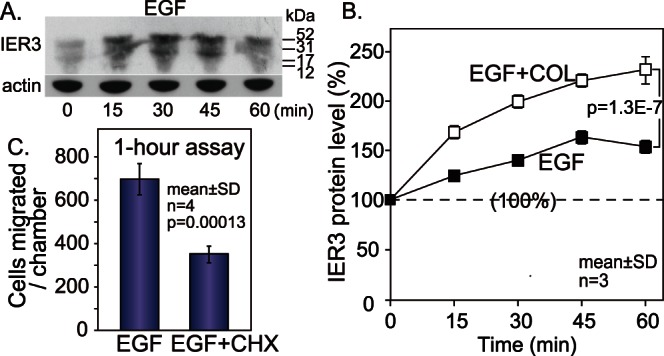
IER3 protein is promptly synthesized in response to EGF and adhesion. (A) Kinetics of IER3 protein expression evaluated by western blotting. (B) Kinetics of IER3 protein expression measured by dot blot analysis. (C) The effect of cycloheximide on early migration of Calu1 cells. Two way analysis of variance was used for statistical analysis.

It was suggested that immediate early response protein3, IER3 (formerly known as IEX1) induced sustained activation of ERK [66] by inhibiting B56-containing PP2A [39]. Indeed, we found, by using a pulldown assay, that IER3 binds to B56γ1 (Figure 4A). IER3 was not coprecipitated with B56α1 and B56β1 by the pulldown assay (Figure 4A). If IER3 induces sustained activation of ERK for cell migration through interaction with PP2A-B56γ1, the IER3 protein should be fully expressed within 30 min after costimulation with EGF plus COL. It has been reported that the expression of IER3 mRNA starts within 10 minutes after EGF stimulation [67], but it is not known exactly how long it takes for IER3 protein expression to begin. We therefore examined the kinetics of IER3 protein expression following stimulation with EGF or EGF+COL. Unglycosylated and glycosylated forms of IER3 were visualized by western blotting (Figure 5A) and dot blot analysis was used for quantification. We found that, when the cells were stimulated with EGF or EGF+COL, IER3 protein was moderately expressed within 15 min (Figure 5B), suggesting that the protein expression started within a few minutes of stimulation. The IER3 protein accumulated more rapidly when the cells were stimulated with EGF+COL than when they were stimulated with EGF alone (Figure 5B). These results led us to re-examine the effect of cycloheximide on EGF-induced migration at an early time point, and we found that cycloheximide inhibited EGF-induced cell migration at 1 hour after EGF administration (Figure 5C).

**Figure 6:**
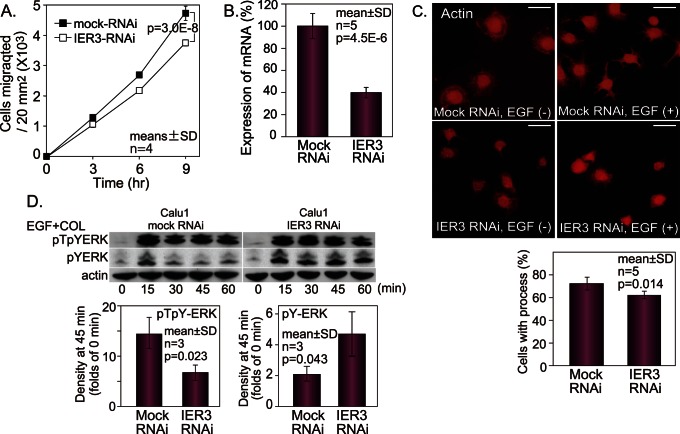
IER3 knockdown decreases cell migration and increase ERK dephosphorylation. (A) The effect of IER3 RNAi on migration of Calu1 cells. (B) The effect of RNAi on IER3 mRNA expression. (C) The effect of IER3 RNAi on the structure of actin cytoskeleton. The numbers of cells with cytoplasmic processes were counted under a fluorescence microscope. (D) The effect of IER3 RNAi on kinetics of pYERK and pTpYERK. Bars, 10 µm. Two way analysis of variance (A) or Student’s t-test (B, C, D) was used for statistical analysis.

We next determined the effect of IER3 knockdown on cell migration. RNAi against IER3 inhibited the migration of Calu1 cells (Figure 6A). The rate of decrease in migration was 20% at 9-hour migration, which was less than the effect of B56γ1 overexpression (Figure 2D), and then the inhibitory efficiency of mRNA expression was measured by a real time PCR. The inhibition of the expression of IER3 mRNA was not complete but 38% (Figure 6B). When Calu1 cells or Calu1 cells transfected with mock d-siRNA were treated with EGF and loaded onto collagen-coated glass slides, the cells exhibited cytoplasmic processes (Figure 6C). When Calu1 cells were transfected with IER3 d-siRNA, generation of these cell processes was inhibited (Figure 6C), indicating that these cells would have low migratory activity [60]. To quantify the difference in the effect between mock RNAi and IER3 RNAi, the numbers of cells with cytoplasmic processes were counted under a fluorescence microscope. There was a significant difference in the percentages of cells positive for cytoplasmic processes (Figure 6B). The effects of IER3 knockdown on ERK phosphorylation were also examined by western blotting using anti-pTpYERK and anti-pYERK antibodies. IER3 knockdown led to enhanced dephosphorylation of pTpYERK, resulting in a change from sustained activation to transient activation of ERK (Figure 6D). In contrast, IER3 knockdown changed the transient increase in pYERK to a sustained increase by enhancing dephosphorylation of pTpYERK at the threonine residue (Figure 6D).

### Kinetics of Activities of B56γ1, ERK and MEK Following EGF/COL Stimulation

**Figure 7:**
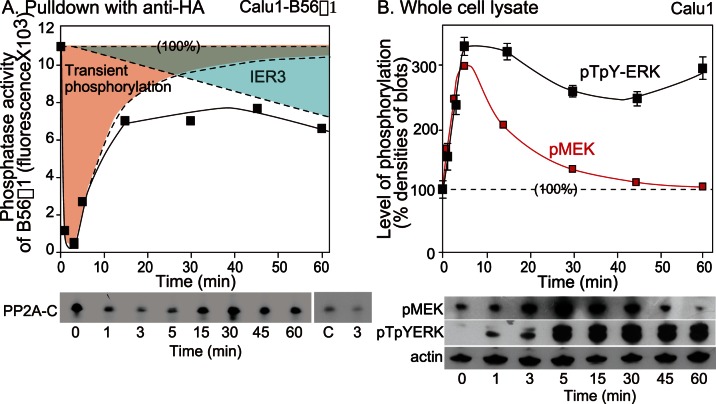
The strength and duration of ERK activation is determined by MEK and PP2A-B56γ1 activities. (A) The activity of PP2A-B56γ1 phosphatase measured by immune complex fluorometric phosphatase assay using 4-methylumbelliferyl phosphate as a substrate. The closed squares indicate the measured phosphatase activity. Two areas demarcated by dotted lines (“transient phosphorylation” and “IER3”) were deduced from the effect of two distinct factors, i.e., the rapid inactivation of phosphatase activity (probably due to phosphorylation of the C subunit of PP2A) and the later PP2A inactivation reaction by IER3. C subunits of PP2A in the immune complex were detected by western blotting using anti-C subunit antibodies. Since faint blot at 3 min shows non-specific binding to Sepharose beads, densities of non-immune controls (C) and of 3 min are being compared. (B) The kinetics of ERK and MEK activities in Calu1 cells were examined by western blotting. The plot was constructed using data from 3 (0 to 15 min) or 9 (0, 15 to 60 min) repeated experiments.

Calu1-B56γ1 cells were stimulated with EGF/COL, and PP2A-B56γ1 phosphatase activity between 0 and 60 min, especially during the first 15 min period, was examined using an immune complex fluorometric assay. Unexpectedly, these data showed that PP2A-B56γ1 activity dramatically decreased within 1 min of stimulation, to the point where no activity could be detected (Figure 7A). However, after a few more minutes, PP2A-B56γ1 activity started to increase, and then it gradually increased over the next 15 min, and was maintained at approximately the same level between 15 min and 60 min (Figure 7A). The recovered activity was lower than the initial activity. The kinetics of phosphatase activity over the entire 60 min after stimulation suggested the existence of two different phases. The earlier phase involves a robust inactivation, and the later phase shows a sustained moderate activation. We next analyzed the presence of the catalytic C subunit of PP2A in the same HA-precipitated immune complexes by western blotting using anti-Csubunit antibodies. The C subunit that was initially bound to B56γ1 was not detected in precipitates between 1 to 5 min after treatment but was detected at subsequent time points and its levels were maintained at an approximately similar level between 15 min and 60 min of stimulation. The time course of the association of the C subunit of PP2A was coincident with the kinetics of PP2A-B56γ1 phosphatase activities (Fig. 7A).

Sustained inactivation of PP2A-B56γ1 phosphatase activity should correspond with sustained phosphorylation of ERK. We therefore reassessed the kinetics of ERK phosphorylation in the later phase following EGF/COL stimulation. The intensities of the bands of pTpYERK previously obtained by western blotting were plotted (Figure 7B). The plot of the kinetics of pTpYERK phosphorylation was the reciprocal of that of PP2A-B56γ1 activity (Figure 7A, B). We also examined the kinetics of pTpYERK phosphorylation in the early phase following EGF/COL treatment (Figure 7B). The highest peak of pTpYERK phosphorylation was at 5 min, which was 4 min after the initiation of PP2A-B56γ1 inactivation. To determine the significance of the inactivation of PP2A-B56γ1 phosphatase activity, we next analysed the kinetics of MEK phosphorylation. It is well established that MEK is a dual kinase that is required for ERK activation. The kinetics of MEK activation was determined by western blotting of the active form of MEK, which is MEK that is phosphorylated at Ser^217/221^ (pMEK), using the same cell lysates as those used for analysis of the early kinetics of pTpYERK. Similar to pTpYERK, the level of pMEK also reached a maximum at 5 min after the start of stimulation. The level of pMEK decreased more rapidly than the level of pTpYERK (Figure 7B). The combined data suggest that inactivation of PP2A-B56γ1 prior to the initiation of MEK and ERK phosphorylation could be involved in transmission of the signal from MEK to ERK.

The subsequent changes in PP2A-B56γ1 activity over time presumably affect the phosphorylation of ERK as follows. After activated MEK has fully phosphorylated ERK at 5 min and pMEK has decreased, ERK phosphorylation would still be increasing as a consequence of overall MEK activity, even if the level was low, unless PP2A-B56γ1 was able to access pTpYERK. The activity of PP2A-B56γ1 at 15 min after stimulation, which had recovered to half the original level of activity at time 0, would decrease the MEK-induced phosphorylation of ERK at 15 min. Indeed MEK phosphorylation had decreased to half the peak value by 15 min. Therefore, the level of ERK phosphorylation would be maintained at the same level due to the balance of the opposing actions of MEK1 and PP2A-B56γ1.

## Discussion

McCright et al. [68] reported that B56α1 and B56β1 are localized in cytoplasm, and that B56γ1 is concentrated in nuclei. Other studies have reported that B56γ1 is localized in focal contacts together with paxillin [46] and also in the Golgi complex [63]. These conflicting data regarding the localization of B56γ1 may have led to misinterpretation of its functions. However, we confirmed here that B56γ1 is localized in nuclei by means of a confocal laser scanning microscopic study using the same anti-mouse B56γ1 antibody and the same animal species as those used by Ito et al. [46], [63]. The reported difference in B56γ1 localization between their work and ours could be due to the presence or absence of stimulation. Furthermore, B56γ1 inhibited migration, formed a complex with pTpYERK, probably together with the A and C subunits of PP2A, and dephosphorylated pTpYERK in cell lysates. Among the results obtained in the present study, we could not detect association of B56γ1 and pTpYERK by immunofluorescence but by pulldown assay. The enzyme-substrate reaction should be prompt as the immunofluorescence could not detect the association. However, the reaction should not have been completed so promptly, and the unreacted substrates could have been observed by the pulldown assay. Thus, B56γ1 functions to regulate nuclear ERK activation through dephosphorylation at the TEY motif.

The combined results of the present study showed that there is an interaction between IER3 and PP2A-B56γ1, which results in IER3-mediated inhibition of the dephosphorylation of pTpYERK, thereby leading to sustained ERK activation in the nucleus and cell migration. Our findings reveal two important aspects of IER3 regulatory function towards B56γ1. First, we showed that IER3 expression started within a few minutes after stimulation. Second, we showed that the kinetics both of sustained ERK activation and augmented IER synthesis were caused by costimulation of EGF and collagen adhesion. Sustained ERK activation is likely to be caused indirectly via the effect of augmented IER3 synthesis on PP2A-B56γ1 activity, since an inhibitory effect of IER3 on PP2A-B56γ1 activity via release of the C subunit has already been reported by Letourneux et al. [39]. The expression of IER3 in costimulated cells was higher than that in the cells stimulated with EGF alone, possibly due to the greater ERK activation. The expression level is high enough to prevent ERK dephosphorylation in conjunction with decreased but still substantial MEK activity, leading to sustained activation of ERK in a positive feedback manner.

The kinetics of PP2A-B56γ1 activity suggested that the observed inactivation of PP2A-B56γ1 phosphatase was caused by two different mechanisms. The later phase of phosphatase inactivation could be due to newly synthesized IER3 (Figure 7A right upper triangle demarcated by dotted lines), which induces sustained activation of ERK leading to cell migration. On the other hand, PP2A-B56γ1 activity was lost rapidly in the earlier phase. Considering that there is a background level of phosphatase activity of PP2A-B56γ1 at 0 min in Calu1 carcinoma cells, one possibility is that the extinction of PP2A-B56γ1 activity in the early phase might be important to achieve stable signalling for induction of ERK phosphorylation. Unless constitutively active PP2A-B56γ1 was inactivated, as found in the present study, ERK phosphorylation would be influenced by PP2A-B56γ1 activity. This situation would result in a decrease in the level of pTpYERK and also in an unstable signal dependent upon background phosphatase activities. Thus, the transient extinction of PP2A may play a passive but extremely important role in ensuring the fidelity of the strength of ERK activation. The rapid inhibition of PP2A-B56γ1 activity might be the same event as previously reported [69], [70]; in that work, Src transiently inactivated the phosphatase activity of PP2A by phosphorylation of the C subunit at Tyr^307^, leading to dissociation of the C subunit from PP2A heterotrimer [70]. However, it was also noted that the peak of inactivation of PP2A was at 15 min after stimulation [70]. If that were the case, the significance of the phenomenon would be totally different from what we suggest here, as they claimed that the transient deactivation of PP2A might enhance transmission of the cellular signal [69]. However, the time lag in their case might have been due to their experimental procedure. If the reported inactivation signal from Src was the same signal as the immediate inactivation of PP2A in the present study, it could have originated both from growth factor receptor and integrin, since it has been reported that a growth factor receptor caused PP2A deactivation through Src [69] and Src is involved in integrin-generated signals [71]. Thus, the immediate robust inactivation of PP2A-B56γ1 could be due to transient phosphorylation/inactivation of PP2A (Figure 7A left area demarcated by dotted lines and a solid line), and the synergistic Src-PP2A-ERK signal should be synchronized to the synergistic MEK-ERK signal from growth factor and integrin-mediated anchorage [16].

The PP2A B56γ gene encodes four differentially spliced forms, PP2A-B56γ1, γ2, γ3 and γ4. Amino acids of the spliced variants are identical until exon 12, and B56γ2, γ3, and γ4 have an additional exon or exons [72], [73]. In regard to a specific binding domain, they all have the same amino acids from 391 to 402, which constitute a domain required for interaction with p53 [49]. Indeed, PP2A-B56γ1, γ2 nd γ3 have been reported to dephosphorylate p53 protein at Ser15 [48], [49], [74]. Similarly, all the γvariants could be plausible to bind to ERK as the present study showed that B56γ1 bound to and phosphorylated ERK. Presumably, B56γ1 have a specific binding site to ERK, which has not been determined yet. In addition, other isoforms of B subunit might phosphorylate ERK in the cytoplasm as PP2AB56γ1 did in the nucleus, since phosphorylated ERK localizes not only in the nucleus but anywhere in the cytoplasm [4].

### Conclusions

**Figure 8:**
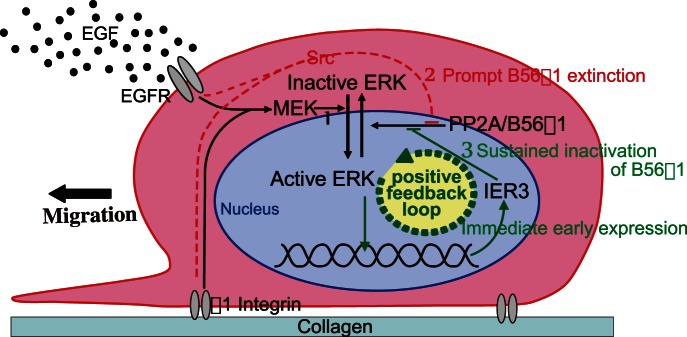
Schematic illustration of proposed mechanisms of ERK-mediated cell migration regulated by PP2A-B56γ1. The numbers 1, 2 and 3 indicate the main signalling pathways that regulate ERK activation within the first 60 min following stimulation.

Our results indicate that the strength and duration of ERK activity are regulated by a combination of MEK and PP2A-B56γ1 activities. We propose that the machinery of the spatio-temporal regulation of ERK is as illustrated in Figure 8. First, MEK activates ERK (Figure 8-1). The ERK activity transmitted by MEK is initially unaffected by B56γ1-PP2A due to its prompt extinction (Figure 8-2), but subsequently the balance of MEK and B56γ1-PP2A activities determines the strength of ERK activation. A positive feedback loop involving IER3-B56γ1-phosphorylated ERK (Figure 8-3) determines the duration of ERK activation.
