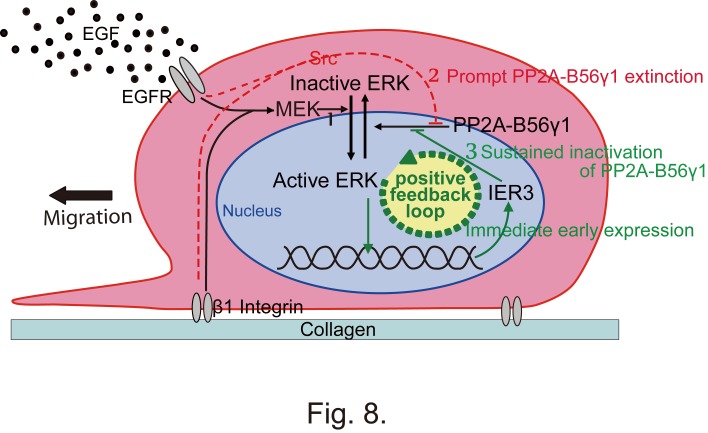# Correction: Dynamic Regulation of Extracellular Signal-Regulated Kinase (ERK) by Protein Phosphatase 2A Regulatory Subunit B56γ1 in Nuclei Induces Cell Migration

**DOI:** 10.1371/annotation/0c13510e-5537-49c0-906f-9cfa842f0363

**Published:** 2013-12-31

**Authors:** Ei Kawahara, Shiori Maenaka, Eri Shimada, Yoshihiro Nishimura, Hiroshi Sakurai

An error occurred in Figures 2, 3, 4, 7, and 8 that prevented the Greek symbols in B56α1, B56γ1, and B56β1 from being displayed. Please see the corrected Figures here:

Figure 2: 

**Figure:**
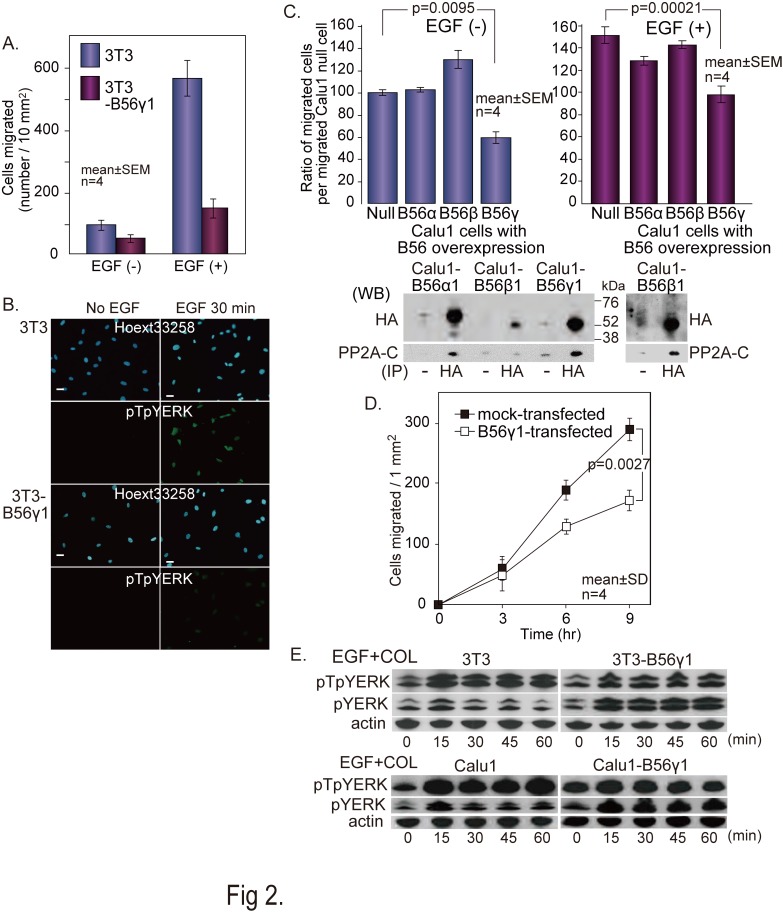


Figure 3: 

**Figure:**
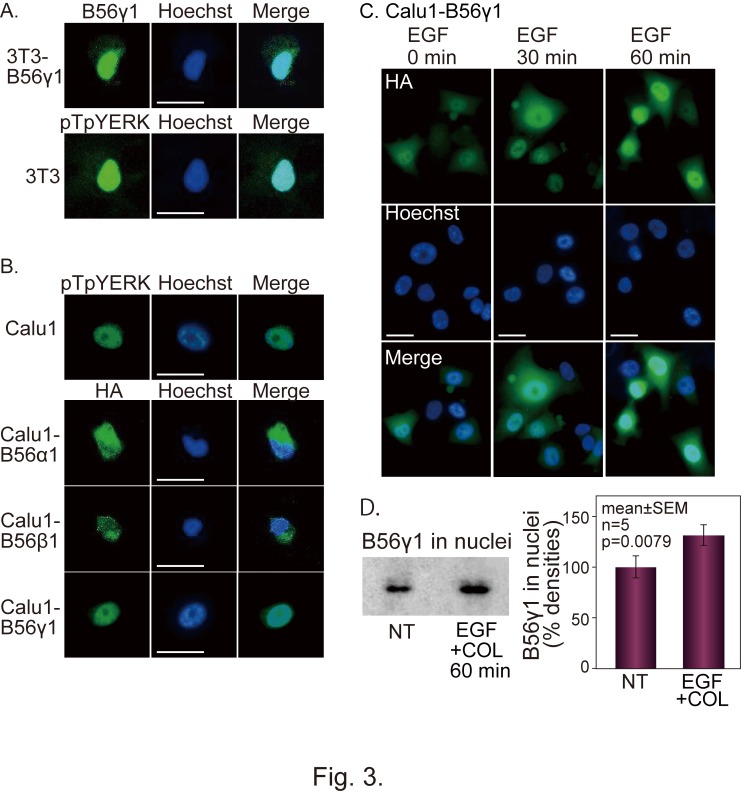


Figure 4: 

**Figure:**
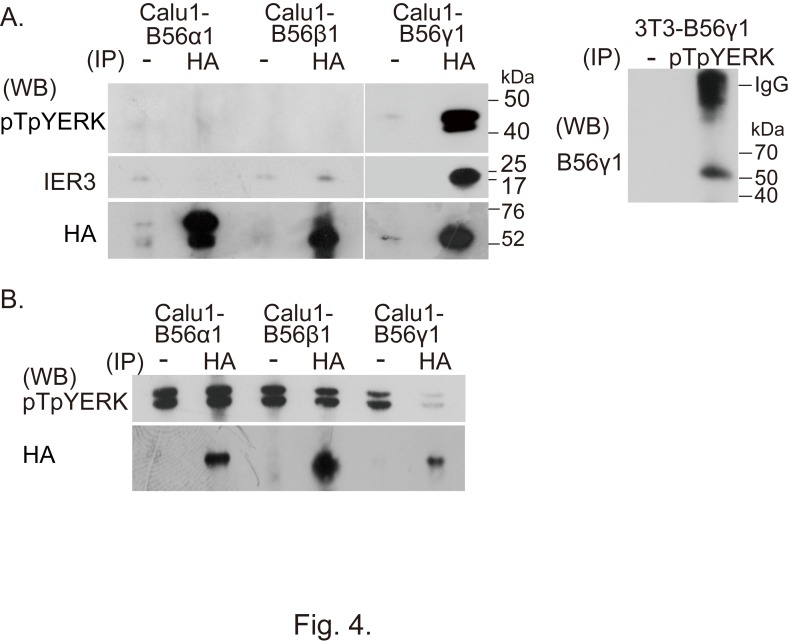


Figure 7: 

**Figure:**
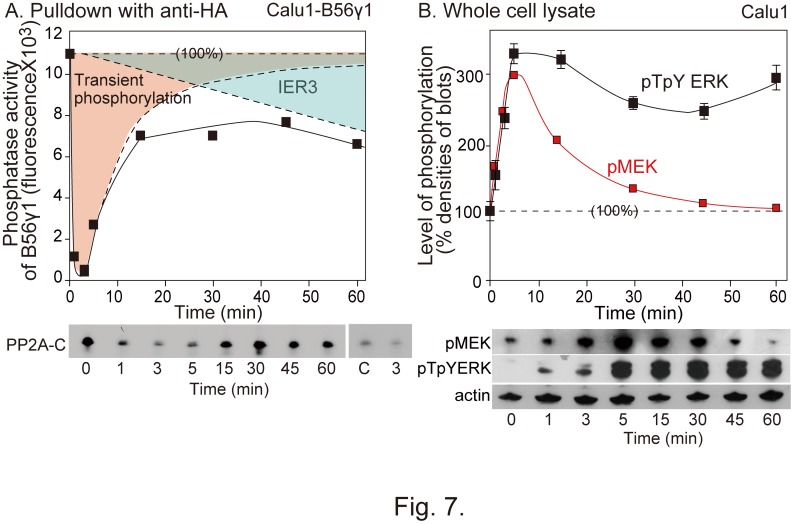


Figure 8: 

**Figure:**